# Exploring implicit and explicit aspects of sense of agency

**DOI:** 10.1016/j.concog.2012.10.005

**Published:** 2012-12

**Authors:** J.W. Moore, D. Middleton, P. Haggard, P.C. Fletcher

**Affiliations:** a Department of Psychology, Goldsmiths, University of London, London SE14 6NW, UK; b Brain Mapping Unit, Department of Psychiatry, University of Cambridge, Cambridge CB2 0SZ, UK; c Institute of Cognitive Neuroscience, University College London, London WC1N 3AR, UK

**Keywords:** Sense of agency, Agency, Voluntary action, Volition, Feeling of agency, Judgement of agency, Dissociation, Learning, Consciousness

## Abstract

Sense of agency refers to the sense of initiating and controlling actions in order to influence events in the outside world. Recently, a distinction between implicit and explicit aspects of sense of agency has been proposed, analogous to distinctions found in other areas of cognition, notably learning. However, there is yet no strong evidence supporting separable implicit and explicit components of sense of agency. The so-called ‘Perruchet paradigm’ offers one of the few convincing demonstrations of separable implicit and explicit learning systems. We adopted this approach to evaluate the implicit–explicit distinction in the context of a simple task in which outcomes were probabilistically caused by actions. In line with our initial predictions, we found evidence of a dissociation. We discuss the implications of this result for theories of sense of agency.

## Highlights

► A distinction between implicit and explicit aspects of agency has been proposed. ► The validity of this distinction is unclear. ► We tested whether these aspects of agency are differentially affected by sequential patterns of action-outcome relations. ► We found evidence for a dissociation between implicit and explicit aspects of sense of agency. ► We discuss the implications of these findings for theories of sense of agency.

## 1 Introduction

Sense of agency refers to the sense of initiating and controlling actions in order to influence events in the outside world. Synofzik, Vosgerau, and Newen (2008) have recently proposed a two-step account that distinguishes between implicit and explicit aspects of sense of agency. At the implicit level, a basic low-level feeling of being an agent is formed. Synofzik and colleagues refer to this as the ‘Feeling of Agency’. This level is non-conceptual, and does not involve explicit agency attributions. Rather, experiences of action are simply tagged as self-caused or not. Although this feeling of agency may be ‘conscious’, it is a *pre-reflective* state (Gallagher, 2000; Synofzik et al., 2008). That is, the feeling of agency does not involve a reflective act of consciousness and is therefore not, in this sense, explicit. It is suggested that this aspect of sense of agency is informed by sensorimotor processes, such as efferent motor information and sensory feedback. At the explicit level, a higher-order conceptual judgement of being an agent is formed. Synofzik and colleagues refer to this as the ‘Judgement of Agency’. At this level, explicit attributions of agency to oneself or another are made. It is suggested that this aspect of sense of agency is closely linked to higher-level sources of information like beliefs, as well as social and contextual cues.

The existence of, and relationship between, these aspects of sense of agency is still a matter for debate and has been the topic of an increasing amount of research on sense of agency (David, Stenzel, Schneider, & Engel, 2011; Miele, Wager, Mitchell, & Metcalfe, 2011; Obhi & Hall, 2011). Analogous distinctions between implicit and explicit representations exist in other areas of cognition, notably learning. Implicit learning is evident in a clear influence of experience on responses in the absence of explicit knowledge or awareness (Cleeremans, Destrebecqz, & Boyer, 1998). For example, in Reber’s early artificial grammar learning study (Reber, 1967), participants were asked to memorise letter strings generated by a finite-state grammar. Following this memorisation stage, participants were told that these letter strings followed a grammatical rule and they would now have to classify new letter strings as grammatical or not. Reber found that whilst participants were able to correctly classify these new strings at a level significantly above chance, they were unable to correctly describe the grammatical rules that generated them. Despite evidence such as this, the distinction between implicit and explicit learning systems remains controversial, with some authors suggesting that the evidence for implicit learning is weak at best (Shanks, 2010).

**Fig. 1:**
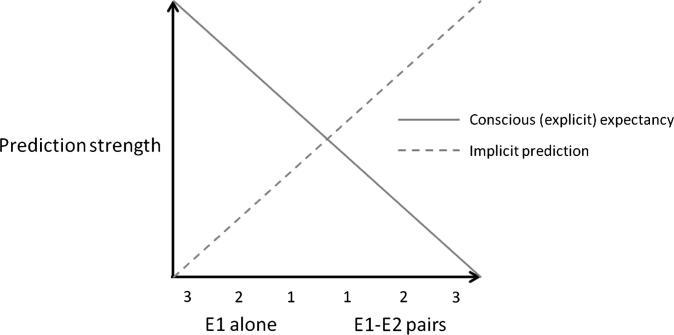
Schematic depicting the dissociation between implicit and explicit measures of learning and prediction (following Perruchet et al. (2006)). Implicit measures show a decrease in prediction strength following longer runs of E1 alone trials and an increase in prediction strength following longer runs of E1–E2 pair trials. On the other hand, explicit measures show an increase in prediction strength following longer runs of E1 alone trials and a decrease in prediction strength following longer runs of E1–E2 pair trials.

A paradigm developed by Perruchet and colleagues offers a rigorous way of experimentally scrutinising this possible dissociation between implicit and explicit learning systems (Perruchet, 1985; Perruchet, Cleeremans, & Destrebecqz, 2006). Briefly, implicit and explicit measures of learning and prediction are pitted against each other in the context of a probabilistic learning task. In this task there is a probabilistic relationship between two events (E1 and E2). After a run of trials in which E1 is followed by E2, explicit measures of learning show a decrease in the strength of E2 prediction, consistent with the gambler’s fallacy. That is, people increasingly expect an event that has not occurred for some time (in this case, E1 in the absence of E2). On the other hand, implicit measures (such as reaction times, Perruchet et al. (2006); or eye-blink conditioning, Perruchet (1985)) show the reverse effect of repeated E1–E2 pairing, namely an increase in the strength of E2 prediction, consistent with the recency effect. Thus, on the critical trials following repeated E1–E2 pairings, explicit judgement suggests that E2 is not predicted, while implicit learning suggests that it is. This dissociation between implicit and explicit measures of prediction (depicted in Fig. 1) provides strong evidence for the existence of separable learning systems.

Previous experimental and theoretical work has highlighted the importance of learning and prediction for sense of agency (Blakemore, Wolpert, & Frith, 2002; Moore & Haggard, 2008). Moreover, at the implicit level, this learning is thought to operate according to basic associative learning principles (Moore, Dickinson, & Fletcher, 2011). Given this, and the putative distinction between implicit and explicit aspects of sense of agency described above, the present study took its inspiration from Perruchet’s dissociation approach. Our implicit measure was based upon the intentional binding paradigm. Intentional binding refers to the compression in the perceived duration of an interval between a movement and its consequence when that movement is under one’s own voluntary control (Haggard, Clark, & Kalogeras, 2002; Moore & Obhi, 2012). In the standard version of this paradigm, participants judge the onset of either voluntary actions (a key press) or the onset of a sensory event (a tone) presented 250 ms after the action. The perceived onset of the action is shifted later in time relative to the perceived onset of actions in a baseline condition in which the action does not produce a tone. Furthermore, the perceived onset of the tone is shifted earlier in time relative to the perceived onset of tones in a baseline condition in which the tone is presented without action. This binding effect is specific to *voluntary* action. When an action is passively induced, participants do not show any binding (in fact, there is a reversal of this effect). On this basis it was suggested that intentional binding is an *implicit* marker of sense of agency. There is now considerable evidence supporting this view (Moore & Haggard, 2010; Moore & Obhi, 2012).

In the present experiment there was a probabilistic relationship between a key press and a tone outcome. Following the standard Perruchet paradigm (e.g. Perruchet et al., 2006), the tone outcome occurred on 50% of trials at random. Because the tone did not always occur we only measured the perceived time of action. We contrasted this implicit intentional binding measure of sense of agency with an explicit one in which participants made a subjective prediction of the likelihood that their action would cause an outcome on the next trial. Many previous agency studies (e.g. Chambon, Wenke, Fleming, Prinz, & Haggard, 2012) have favoured retrospective agency judgements (such as asking participant to rate the extent to which they felt their action caused the previous event). However, we felt this was inappropriate given the absence of another possible cause of the tone. We compared these implicit and explicit indices of sense of agency as a function of the nature and length of preceding action-outcome trials. Based on the logic of the ‘Perruchet paradigm’, if there are separate independent agency processing systems we would expect a dissociation between these implicit and explicit agency measures. That is, explicit prediction that a tone will follow an action should be high after a run of ‘action only’ trials and low after a run of ‘action + tone’ trials (in line with the gambler’s fallacy). On the other hand, intentional binding should be strong after a run of ‘action + tone’ trials and weak after a run of ‘action only’ trials (following the principles of associative learning). In this way, we predicted a significant negative linear trend for explicit prediction and a significant positive linear trend for intentional binding. Furthermore, Perruchet et al. (2006) additionally observed a significant cubic trend for the explicit prediction measure. This was due to the presence of a ‘positive recency effect’, whereby explicit prediction after a single E1 event is *lower* than after a single E1–E2 pairing (see also, Hyman, 1953). In line with this we also predicted a significant cubic trend for our explicit prediction measure only.

## 2 Methods

### 2.1 Participants

Twenty-three participants were initially recruited to the study. Three were subsequently excluded; one on the basis of failure to follow task instructions, one on the basis of uncorrected visual impairment (self-reported by participant) and one due to experimenter error. Of the final sample of 20 participants, all were right-handed, 12 were female and the average age was 27.5 years (range: 21–36 years).

### 2.2 Procedure

**Fig. 2:**
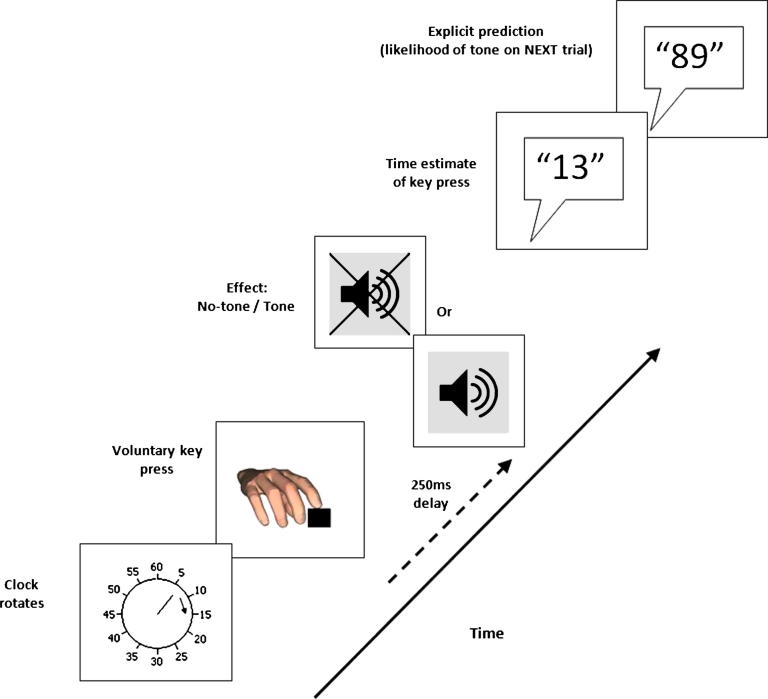
Trial structure in the *operant condition*. At the start of each trial the clock-hand rotated around the clock-face (and would do so until the end of the trial). Whenever they felt the ‘urge’ participants would press a key with their right index finger. On 50% of trials this would cause a tone. The order of tone presentation was randomised. At the end of the trial the participant would report the time of their key press (implicit prediction) and also estimate the likelihood of the tone on the next trial (explicit prediction).

On each trial in the *operant condition* participants pressed a key whenever they felt the urge to do so. They were instructed to press as spontaneously as possible and to refrain from pre-planning their movements. On 50% of trials selected at random, this key press caused a tone, which was presented after a short 250 ms delay. At the same time a clock hand rotated rapidly around a clock face presented on a computer screen in front of them. The clock hand completed one revolution every 2.56 s. The clock hand continued to rotate for a random period of time after the key press. On each trial, participants had to judge the onset of their key press by reporting the position of the clock hand when they depressed the key. Next, participants were prompted to ‘Rate the extent to which you believe there will be a tone on the NEXT trial’. They indicated this by reporting a number between 0 (‘Definitely no tone’) and 100 (‘Definitely a tone’). The schematic in Fig. 2 depicts the trial structure. There were 300 trials in total, split into 6 blocks of 50 trials (allowing regular periods of rest). Because the tone did not always occur we focussed only on the perceived time of the action.

Each participant also performed a *baseline condition* for the intentional binding task. Participants pressed a key whenever they felt the urge to do so, but this key press never caused a tone. In this condition, participants reported the time they pressed the key (because there were no tones, no explicit predictions were collected). There was a single block of 50 trials. Half the participants completed the baseline condition at the start of the experiment and half at the end.

### 2.3 Data analysis

Following Perruchet et al. (2006), each trial was classified according to the nature and length of the preceding trial runs. Specifically, trials were classified according to whether the preceding trial consisted of a run of 1, 2 or 3 ‘Action only’ trials or a run or 1, 2 or 3 ‘Action + tone’ trials (see Table 1 for details).

For the intentional binding measure we analysed shifts in the perceived time of action. This shift was calculated as the judgement error in the *operant condition* minus judgement error in the *baseline condition*. These shifts were analysed as a function of the nature and length of the trials that preceded it. For the explicit prediction measure we analysed explicit predictions as a function of the same information. It is important to point out what determines the starting point of previous learning history for the two different measures (i.e. trial n-1) relative to each individual trial in the experiment. For explicit prediction, the start point is the current trial. This is because on each trial the learning event (‘Action only’ or ‘Action + tone’) occurs prior to the prediction being made. For intentional binding, previous learning history starts from the *previous* trial. This is because on the current trial the learning event (‘Action only’ or ‘Action + tone’) happens *after* the participant’s action.

## 3 Results

**Fig. 3:**
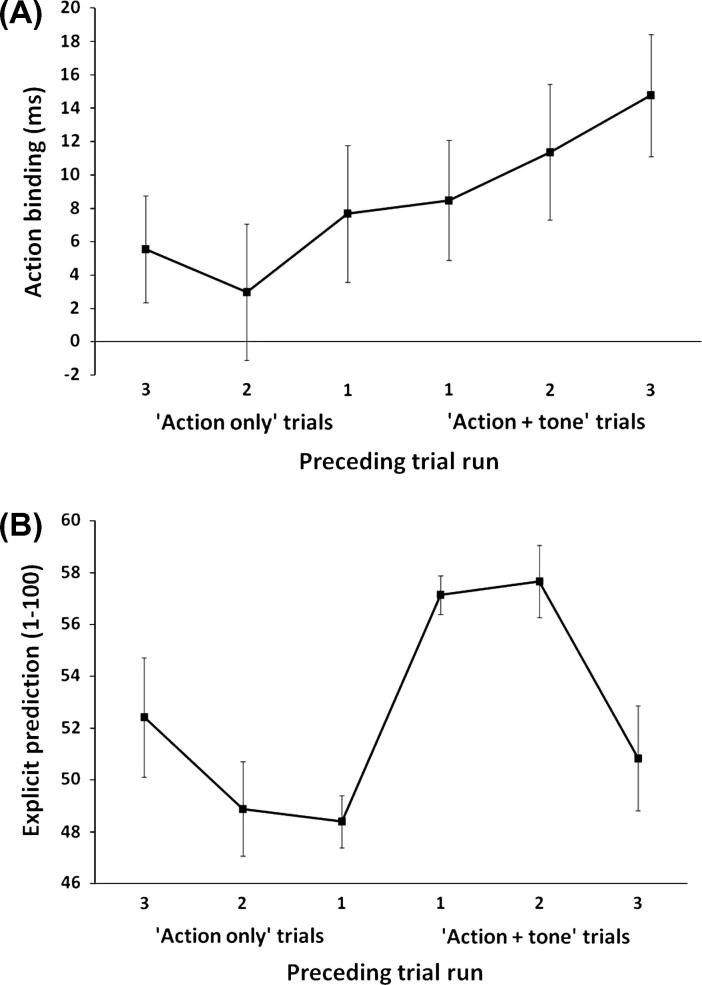
(A) Action binding plotted as a function of learning history. (B) Explicit predictions (1 = ‘definitely no tone’/100 = ‘definitely a tone’) plotted as a function of learning history. Error bars represent standard error of the mean. Action binding refers to the difference in judgement error in the operant vs. baseline conditions. The more positive the difference, the larger the action binding effect.

Fig. 3A and B show the results for intentional binding and explicit prediction respectively. As can be seen, the nature and length of preceding trial runs had different effects on intentional binding and explicit prediction. This was supported by statistical analyses. Separate trend analyses were performed for intentional binding and explicit prediction measures, with ‘preceding trial run’ as a within-subjects factor (‘Action only’ trials: 3, 2, 1; ‘Action + tone’ trials: 1, 2, 3).

For intentional binding there was a significant main effect of ‘preceding trial run’, *F*(5, 95), 2.37, *p* = .045, $\nu_{\text{partial}}^{2}=.11$. More importantly, and as predicted, for intentional binding there was a significant positive linear trend, *F*(1, 19) = 5.96, *p* = .03, $\nu_{\text{partial}}^{2}=.24$, indicating a stronger action-outcome association following longer runs of operant trials. Also as predicted, there was no significant cubic trend for the intentional binding measure, *F*(1, 19) = .22, *p* = .65, indicating the lack of a positive recency effect.

For explicit prediction the pattern of results was more complicated. Overall, there was no significant main effect of ‘preceding trial run’, *F*(5, 95) = 1.56, *p* = .18. More important were the trend analyses. The strength of prediction decreased with longer runs of ‘action + tone’ trials and increased with longer runs of ‘action only’ trials. This is largely consistent with the gambler’s fallacy. However, contrary to our initial prediction there was no consistent linear decrease for explicit prediction, *F*(1, 19) = .23, *p* = .64. We also predicted a significant cubic trend for explicit prediction (following Perruchet et al. (2006)), indicating a positive recency effect. This was confirmed by our trend analyses, *F*(1, 19) = 19.96, *p* < .001, $\nu_{\text{partial}}^{2}=.51$.

## 4 Discussion

In this experiment we investigated implicit and explicit aspects of sense of agency, and the relation between them. Previous theoretical work has proposed separable implicit and explicit agency processing systems (Synofzik et al., 2008). To test this, we used a method previously developed by Perruchet, which provides one of the more convincing demonstrations of separable implicit and explicit learning systems. Specifically, we explored how intentional binding and explicit prediction of action outcomes varied as a function of the nature and length of previous trial runs. We found a pattern of results that is largely consistent with the Perruchet effect. Based on the logic of this paradigm, our results provide evidence in favour of separable implicit and explicit agency processing systems.

The pattern of results for intentional binding implies an automatic strengthening of association with more repeated operant experience. This finding is consistent with the notion that intentional binding is linked to lower level implicit aspects of sense of agency, mediated by automatic associative learning mechanisms (Moore, Dickinson, & Fletcher, 2011; Synofzik, Vosgerau, & Newen, 2008). Our findings also shed light on the *nature* of the relationship between implicit and explicit aspects of sense of agency. The different pattern of results for implicit and explicit measures suggests that these systems are, at least to some extent, independent. At first glance, this appears to cast doubt on the validity of using intentional binding to probe higher-order aspects of sense of agency, as has been previously suggested (Moore & Haggard, 2010). However, the specific pattern of results might suggest that these two aspects of sense of agency are not fully independent. The cubic trend in our explicit prediction data indicates a ‘positive recency’ effect. This effect has also been observed for explicit measures in previous studies using the Perruchet paradigm (e.g. Perruchet et al., 2006), and is expected given an automatic strengthening of association following the pairing of two-events. Such a pattern of results might suggest that the implicit agency processing system influences the explicit one, albeit on a very short-term basis of the immediately preceding trial. Thus, in the short term the two measures may be partly interdependent. This interdependency may reflect the fact that we took the two measures concurrently, thus increasing the likelihood that one system affects the other (Perruchet et al., 2006). In light of this we would suggest that, whilst implicit and explicit aspects of sense of agency are indeed separable, they are unlikely to be fully independent. This fits the theoretical framework outlined by Synofzik et al. (2008) which emphasises the interaction between these aspects of sense of agency via top-down and bottom-up mechanisms.

In summary, adopting an established dissociation paradigm we explored the putative existence of, and relationship between implicit and explicit aspects of sense of agency. Based on the logic of this paradigm, our results suggest that there are separable, and to some extent independent, agency processing systems. We would suggest that paradigms developed in the context of the implicit and explicit learning debate will be useful for further clarifying the nature of agency processing.

## Figures and Tables

**Table 1:** Classification scheme for the previous three trials (n-1, n-2 and n-3) based on Perruchet et al. (2006).

n-3 Trial	n-2 Trial	n-1 Trial	Trial ‘n’ classification (based on Perruchet et al. (2006))
*‘Action only’ trials*			
‘Action only’	‘Action only’	‘Action only’	3
‘Action + tone’	‘Action only’	‘Action only’	2
‘Action + tone’	‘Action + tone’	‘Action only’	1
‘Action only’	‘Action + tone’	‘Action only’	1

*‘Action + tone’ trials*			
‘Action only’	‘Action only’	‘Action + tone’	1
‘Action + tone’	‘Action only’	‘Action + tone’	1
‘Action only’	‘Action + tone’	‘Action + tone’	2
‘Action + tone’	‘Action + tone’	‘Action + tone’	3
